# The conservation value of cacao agroforestry for bird functional diversity in tropical agricultural landscapes

**DOI:** 10.1002/ece3.5021

**Published:** 2019-06-25

**Authors:** Joedison Rocha, Rudi Ricardo Laps, Caio Graco Machado, Sofia Campiolo

**Affiliations:** ^1^ Programa de Pós‐graduação em Ecologia e Conservação da Biodiversidade Universidade Estadual de Santa Cruz Ilhéus Brazil; ^2^ Laboratório de Ecologia Universidade Federal de Mato Grosso do Sul Campo Grande Brazil; ^3^ Laboratório de Ornitologia – Sala 03, LABIO Universidade Estadual de Feira de Santana Feira de Santana Brazil

**Keywords:** biodiversity hotspot, functional homogenization, functional redundancy, habitat filters, seed dispersal, specialist loss, *Theobroma cacao*, wildlife‐friendly landscape

## Abstract

Cacao agroforestry have been considered as biodiversity‐friendly farming practices by maintaining habitats for a high diversity of species in tropical landscapes. However, little information is available to evaluate whether this agrosystem can maintain functional diversity, given that agricultural changes can affect the functional components, but not the taxonomic one (e.g., species richness). Thus, considering functional traits improve the understanding of the agricultural impacts on biodiversity. Here, we measured functional diversity (functional richness‐FD, functional evenness‐FEve, and functional divergence‐Rao) and taxonomic diversity (species richness and Simpson index) to evaluate changes of bird diversity in cacao agroforestry in comparison with nearby mature forests (old‐growth forests) in the Brazilian Atlantic Forest. We used data from two landscapes with constraining areas of mature forest (49% Una and 4.8% Ilhéus) and cacao agroforestry cover (6% and 82%, respectively). To remove any bias of species richness and to evaluate assembly processes (functional overdispersion or clustering), all functional indices were adjusted using null models. Our analyses considered the entire community, as well as separately for forest specialists, habitat generalists, and birds that contribute to seed dispersal (frugivores/granivores) or invertebrate removal (insectivores). Our findings showed that small cacao agroforestry in the forested landscape sustains functional diversity (FD and FEve) as diverse as nearby forests when considering the entire community, forest specialist, and habitat generalists. However, we observed declines for frugivores/granivores and insectivores (FD and Rao). These responses of bird communities differed from those observed by taxonomic diversity, suggesting that even species‐rich communities in agroforestry may capture lower functional diversity. Furthermore, communities in both landscapes showed either functional clustering or neutral processes as the main driver of functional assembly. Functional clustering may indicate that local conditions and resources were changed or lost, while neutral assemblies may reveal high functional redundancy at the landscape scale. In Ilhéus, the neutral assembly predominance suggests an effect of functional homogenization between habitats. Thus, the conservation value of cacao agroforestry to harbor species‐rich communities and ecosystem functions relies on smallholder production with reduced farm management in a forested landscape. Finally, we emphasize that seed dispersers and insectivores should be the priority conservation targets in cacao systems.

## 1 INTRODUCTION

Habitat change due to agricultural intensification is one of the main drivers of biodiversity loss in tropical forests (Gibbs et al., 2010). Hence, these impacts have created landscapes dominated by different agrosystems, where reforestation actions may be difficult and costly for the entire landscape (Lamb, Erskine, & Parrotta, 2005; Vandermeer & Perfecto, 2007). This underlines a need for new conservation strategies in order to understand and protect species and ecosystem functions in these degraded areas (Lamb et al., 2005; Melo, Arroyo‐Rodríguez, Fahrig, Martínez‐Ramos, & Tabarelli, 2013). There is increasing evidence that the maintenance of biota in tropical landscapes strongly depends on the complexity and composition of cultivated areas, as well as of the proportion of intact habitats in the agricultural systems (Fahrig et al., 2011; Vandermeer & Perfecto, 2007). In this context, the concept of “biodiversity‐friendly landscapes” has been suggested as a potential solution to resolve the conflict between biological conservation and agricultural production (Melo et al., 2013).

The dominance of intensive systems such as monocultures may not support food security and biodiversity conservation together (Melo et al., 2013). However, biodiversity‐friendly farming such as shaded agroforestry systems has been highlighted as key environments for conservation and economic efforts (Bhagwat, Willis, Birks, & Whittaker, 2008). These environments show lower management intensity and, therefore, can harbor high species richness and greater habitat heterogeneity at the landscape scale (Bhagwat et al., 2008). Although agroforestry can indeed harbor greater diversity than monocultures (Barrios et al., 2017), studies have shown that these agrosystems may be associated with declines or changes in communities of forest specialists, plants, terrestrial invertebrates, and birds compared with surrounding forests (Barrios et al., 2017; Bhagwat et al., 2008; Van Bael, Bichier, Ochoa, & Greenberg, 2007). Therefore, these systems are not substitutes for intact forests but may contribute to species conservation at the landscape scale. Furthermore, the impacts of agroforestry on biodiversity rely on the type of crop and the management regime (Jose, 2009). Among these agrosystems, cacao plantations are well‐known examples of a “wildlife‐friendly” type of farming in tropical landscapes (Rice & Greenberg, 2000; Schroth et al., 2011; Van Bael et al., 2007).

Studies in cacao plantations have shown varying proportions of species similarity compared with the nearby forests: 6%–54% for plants, 4%–98% for invertebrates, and 46%–91% for vertebrates (see Bhagwat et al., 2008 for a brief review). These patterns of species composition in cacao agroforestry may be explained by the suppression of understory and midstory vegetation, canopy thinning, and intense management (Rice & Greenberg, 2000). In fact, these structural changes of habitat have shown little effect on some species, while drastically affecting others. For instance, several studies have shown a decrease in understory bird species, but not for species in the canopy (Faria, Laps, Baumgarten, & Cetra, 2006; Van Bael et al., 2007). Despite this, a positive impact of shaded cacao systems for birds, that is, a total richness similar or higher to nearby forests, is often reported (Bhagwat et al., 2008; Faria et al., 2006; Reitsma, Parrish, & McLarney, 2001). However, specific impacts on some functional groups (e.g., understory species) may suggest greater changes in functional diversity, while the species richness is weakly affected. In fact, several studies have shown a less predictive power of species diversity for a complete understanding of landscape effects in tropical and temperate environments (Cadotte, Carscadden, & Mirotchnick, 2011; Flynn et al., 2009; Lindenmayer et al., 2015). Thus, it is necessary to have a better understanding of the importance of cacao plantations in conservation strategies for different facets of biodiversity.

Cacao agroforestry is an important land‐use in coastal northeastern Brazil, especially in southern Bahia. This area represents the central region of cacao production of the country, which overlaps with an important and threatened biodiversity hotspot, the remnants of the Atlantic Forest (Faria, Paciencia, Dixo, Laps, & Baumgarten, 2007). The agroforestry of this region is known as “cabrucas,” which are implemented by understory suppression to establish the cultivation under the thinned canopy of the native forest (Sambuichi, 2006). Despite these habitat simplifications, studies have also shown a relevant conservation value of the cacao plantations for animals and plants in southern Bahia (Faria et al., 2006; Pardini et al., 2009; Sambuichi, 2006). However, these agroforestry do not support all forest specialist species, as well as frugivores and understory insectivores compared with nearby forests (Faria et al., 2006; Pardini et al., 2009). This is the case for birds, which may suggest that the impacts of agroforestry on this group may not be perceived by only using general taxonomic metrics. This re‐emphasizes the need for an approach that takes into account other facets of biodiversity, such as a functional one (Devictor et al., 2010).

The functional diversity approach emerges as an alternative by considering biodiversity traits that affect ecosystem functioning (Cadotte et al., 2011). Given the complexity of this approach, several metrics have been proposed (see Schleuter, Daufresne, Massol, & Argillier, 2010 for a review). Thus, it is currently recognized that functional diversity comprises three complementary components; functional richness, functional evenness, and functional divergence (Mouchet, Villéger, Mason, & Mouillot, 2010). These metrics are required to measure different facets of the functional space, that is, a multidimensional space constrained by traits (axes) where species are placed according to their functional contributions (Mouillot, Grahan, Villéger, Mason, & Bellwood, 2013). Changes in this functional space across disturbance gradients may, therefore, reveal changes in resource use, niche occupancy, and thus ecological functions, not evident from changes in species richness alone (Cadotte et al., 2011; Mason, Mouillot, Lee, & Wilson, 2005). Furthermore, these functional indices may also provide insights into the processes limiting community membership after biological disturbances (Mouillot et al., 2013), including the effects of biotic interactions (or limiting similarity), niche filtering (or environmental filters) (Mouchet et al., 2010), and neutral dispersal and extinction (Hidasi‐Neto, Barlow, & Cianciaruso, 2012). Thus, using multi‐traits functional indices may be a helpful approach to assess the ecological impacts of agricultural activities.

Here, we evaluated the impact of cacao agroforestry on bird taxonomic and functional diversity in southern Bahia, Brazil. Birds provide an important study group because of their central role in key ecosystem processes, including predation and seed dispersal (Whelan, Wenny, & Marquis, 2008). Furthermore, the availability of information on both their responses to habitat change and functional traits make them a tractable system. Our study aims to investigate the following: (a) if the functional diversity of avian communities in cacao agroforestry is lower than that observed in mature forests; (b) if these decreases are the same as observed by taxonomic metrics; and finally, (c) which assembly processes (i.e., limiting similarity or niche filtering) better explain the functional structures of our communities in both habitats. Given that cacao agroforestry are likely to represent a simplified habitat in terms of vegetation structure and resource diversity, we hypothesize that stronger niche filtering in these disturbed environments leads to declines in functional diversity (regardless of species richness) compared with forests, particularly for forest specialists, seed dispersers, and invertebrate‐eaters for which declines in species richness in agroforestry systems are often reported (Faria et al., 2006; Rice & Greenberg, 2000; Van Bael et al., 2007).

## 2 MATERIALS AND METHODS

### 2.1 Study area and species lists

**Figure 1:**
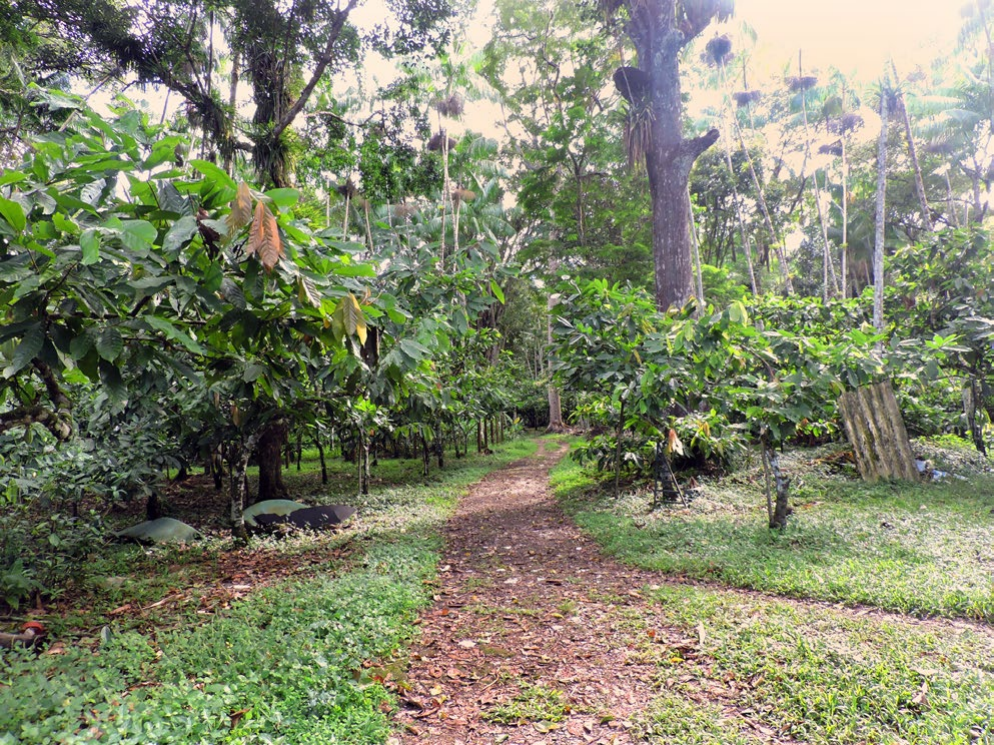
Cacao agroforestry (*cabruca* system) in the municipality of Ilhéus, southern Bahia (Brazil). Photo: Joedison Rocha

**Figure 2:**
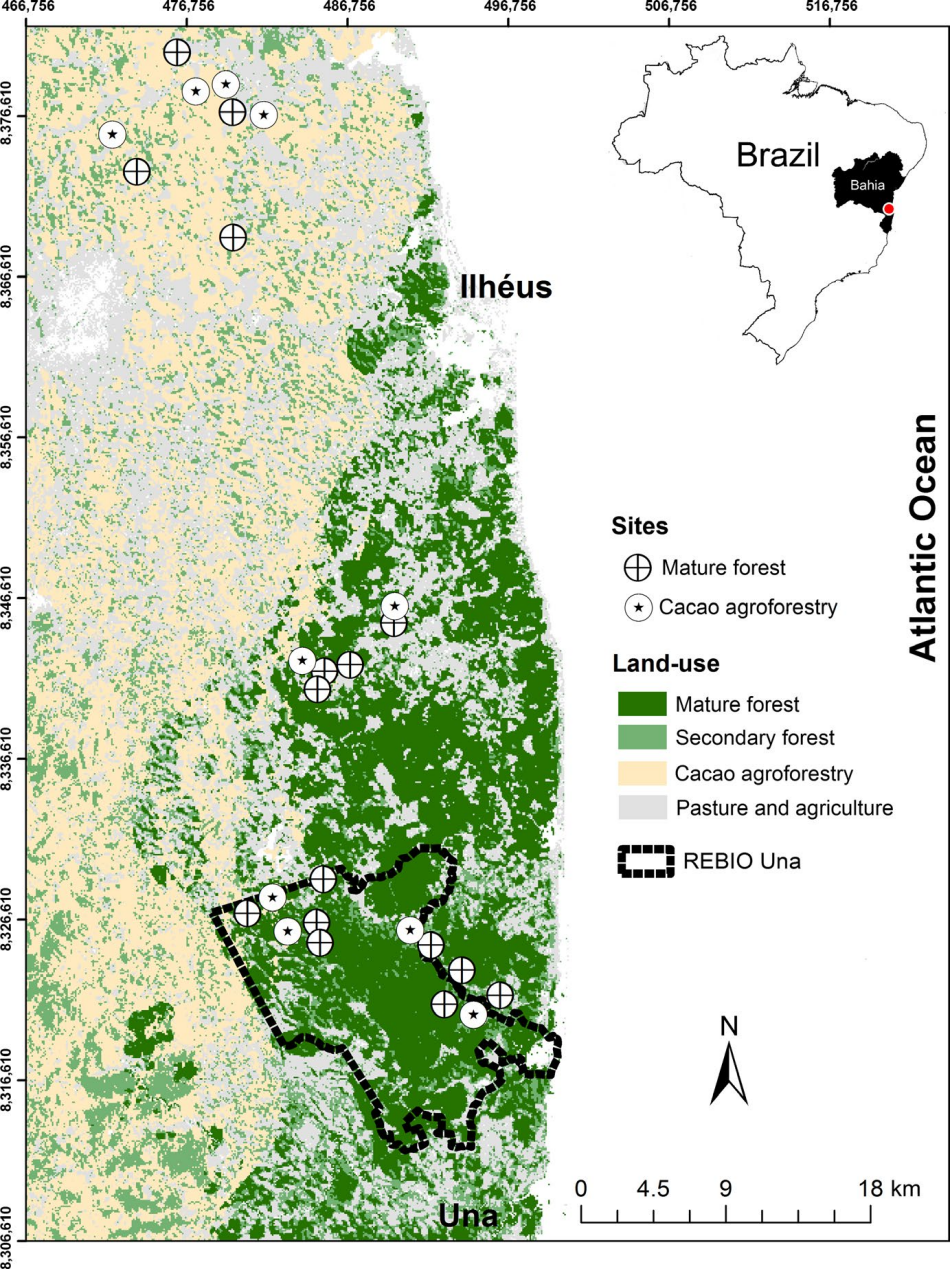
Sampling sites surveyed during the RestaUna Project (1998–2002) and used in this study consisted of 10 cacao agroforestry sites and 16 mature forests sites in the landscape of Una (bottom of the map) and Ilhéus. We also highlight the current perimeter of the Una Biological Reserve (REBIO Una) (modified after Landau, Hirsch, & Musinsky, 2003b; Faria et al., 2006, 2007)

We studied two distinct landscapes in southern Bahia, considered the cacao centre of Brazil (Schroth et al., 2011). Despite having undergone extensive fragmentation, this area is considered important to the global conservation of the Atlantic Forest due to its high richness of rare, endemic and threatened species (Landau, Hirsch, & Musinsky, 2003a). Data from 1996 to 1997 showed that agricultural systems (e.g., cotton, coffee, sugarcane, and rubber tree) plus pastures were the main land‐use of the region, followed by shaded cacao plantations (“cabrucas”; Figure 1), especially in the northern portion of the region (Landau et al., 2003a; Figure 2). Although cacao agroforestry was responsible for part of the degradation process of the Atlantic Forest, it also maintained native forest cover through the preservation of the shade trees beneath which the crop is grown. The composition of the shading trees of these systems varies between native trees from the thinned forest and exotic species such as *Erythrina* spp. (Fabaceae), but native trees are the most common (Faria et al., 2006). In this study, we compiled 26 bird lists (10 in the agroforestry sites and 16 in the mature forest sites) surveyed in southern Bahia during the RestaUna Project between 1998 and 2002 (Table 1; Figure 2; Faria et al., 2006, 2007; Pardini et al., 2009). This survey used standardized sample effort and is one of the few datasets available on bird communities from the cacao agroforestry system of Brazil. Exotic bird species and species inventories compiled from fragment edges were not considered in this study.

**Table 1:** Bird species richness for the overall community and for all ecological subgroups that we tested in this study in the two landscapes of southern Bahia (Brazil) surveyed during the RestaUna Project (1998–2002)

Landscape	Tested groups	Mean richness (*SD*)		*p*
		Forest	Agroforestry	
Una	ALL	73.91 (5.85)	90.50 (7.86)	**<**0.001
	SPE	22.16 (3.37)	15.00 (2.45)	0.001
	GEN	7.83 (2.72)	19.83 (2.78)	<0.001
	FGr	45.75 (4.49)	58.33 (7.78)	<0.001
	INV	67.91 (5.01)	84.00 (6.84)	<0.001
Ilhéus	ALL	32.75 (4.03)	37.95 (4.27)	0.201
	SPE	8.50 (3.41)	4.00 (1.41)	0.083
	GEN	6.50 (1.00)	11.25 (2.62)	0.027
	FGr	19.00 (2.94)	26.00 (2.70)	0.027
	INV	30.50 (3.00)	34.00 (5.16)	0.374

Notes

These landscapes comprise fragments of mature forests (Forest) and cacao agroforestry sites (Agroforestry).

ALL: all birds of the communities; FGr: species that contribute to seed dispersal (frugivores/granivores); GEN: forest generalists; INV: species that contribute to invertebrate removal (insectivores); SPE: forest specialists.

The first studied landscape is located in the municipality of Una (central point: UTM 484973, 8325906, 24S, datum SAD69), where the Una Biological Reserve (18.715 ha) is one of the major fragments of native forests remaining in this area (Figure 2). This landscape is constituted by 49% mature forests (i.e., old‐growth forests), 27% pastures, and 15% secondary forests in the initial stage of regeneration, and 6% cacao agroforestry (Pardini et al., 2009). The second landscape is located in the municipality of Ilhéus, (UTM 473698, 8373196). This is predominantly composed of cacao agroforestry (~82%) and few forest remnants (4.8%) with sizes ranging from 1 to 300 ha (Faria et al., 2006).

In both landscapes, birds were surveyed using point counts, that is, sampling points arranged along transects with a distance of 200 m between each of them, and a sampling time of 15 min. In Una 360 points were surveyed totaling 90 hr of sampling effort, while in Ilhéus 120 points were surveyed totaling 30 hr of sampling effort. We considered sites inside of large mature forest fragments (>1,000 ha, *n* = 6), small mature forests (<100 ha, *n* = 6), and cacao agroforestry (all <100 ha) for Una. In contrast, all agroforestry of Ilhéus (*n* = 4) were large and all forest fragments (*n* = 4) were small (except one with an area of about 300 ha). Further information about the study area, bird lists, and the sample design of landscapes are available in Faria et al. (2006), Faria et al. (2007), and Pardini et al. (2009).

### 2.2 Functional traits

We selected 26 functional foraging traits, of which 25 are binary variables and one is continuous (Table 2). These traits measure how birds use resources in the environment (Whelan et al., 2008) by evaluating resource quantity (i.e., body mass), food types (e.g., invertebrates), food acquisition strata (e.g., canopy), foraging time activity, and behavioral strategies (e.g., gleaning and mixed flock) (see Table 2 for the full list and description of traits).

**Table 2:** Birds traits used in this study to measure bird functional diversity

Trait type	Trait	Scale and categories
Resource quantity	1. Mean body mass (range: 2−2,172 g)	Continuous
Food types	2. Invertebrates	Binary
	3. Fruits	Binary
	4. Seeds and grains	Binary
	5. Nectar	Binary
	6. Flowers	Binary
	7. Foliage, roots, and tubers	Binary
	8. Vertebrates	Binary
Foraging strata/substrate	9. Canopy	Binary
	10. Midstory	Binary
	11. Understory	Binary
	12. Ground	Binary
	13. Water	Binary
	14. Mud	Binary
	15. Air	Binary
Foraging method	16. Pursuit	Binary
	17. Gleaning	Binary
	18. Pouncing	Binary
	19. Pecking	Binary
	20. Grazing	Binary
	21. Scavenging	Binary
	22. Probing	Binary
	23. Hawking	Binary
Activity period	24. Diurnal	Binary
	25. Nocturnal	Binary
Mixed flock	26. Yes	Binary

Note

All categorical traits are binary variables (0 or 1), and the trait “mixed flock” considers whether the bird species participate in mixed flocks or not.

The traits matrix (species by traits) was obtained from information in the literature. For species mean body mass, we used Ramirez, Diniz‐Filho, and Hawkins (2008) and compiled data from the ornithological collection of the Zoology Museum of the Feira de Santana State University (MZFS, Brazil). For the other traits except for the “mixed‐flock” trait, we mainly used Del Hoyo, Elliot, and Sargatal (1992–2002), Del Hoyo, Elliot, and Christie (2003–2006) and Stotz, Fitzpatrick, Parker, and Moskovits (1996). Furthermore, we also compiled data from an additional 83 references listed in Supporting Information Appendix S1. For 29 data‐deficient species (7.12%), we used information from either congeners or the entire family. Finally, the classification of bird species that participate or not in mixed flocks (an important behavioral characteristic of many birds in order to improve resource acquisition) was defined from studies conducted in the Atlantic Forest (Supporting Information Appendix S1) and through consultation with a research specialist on this issue (CG Machado, pers. comm.).

### 2.3 Community and functional diversity indices

We compiled species richness and abundance from the original survey data, and then, we used the Simpson index as an abundance‐based taxonomic measure. This metric is equivalent to the Rao index (described below) without considering functional distances (De Belo, Lavergne, Meynard, Leps, & Thuiller, 2010). With regards to functional diversity, we calculated the following three metrics: Functional Diversity (FD; Petchey & Gaston, 2006), Functional Evenness (FEve; Villéger, Mason, & Mouillot, 2008), and Rao's quadratic entropy (Rao; Rao, 1982; Botta‐Dukát, 2005). To calculate these indices, we converted the compiled trait matrix into a distance matrix by means of the Gower dissimilarity measure, because this metric allows the simultaneous use of categorical and continuous traits with different weights (Podani & Schmera, 2006).

FD consists of the total branch lengths connecting all species in a functional dendrogram, but not considering the segments that connect them at the root of the tree (Petchey & Gaston, 2006). This index was calculated using the function “treeheight” from the vegan R package (Oksanen et al., 2015). All dendrograms were created with the algorithm UPGMA, which is considered the most robust for functional classifications (Podani & Schmera, 2006). FEve measures the regularity of species abundances in the occupied functional space (Villéger et al., 2008) and was calculated using the R function “dbFD” (FD package; Laliberté, Legendre, & Shipley, 2014). This function considers PCoA axes as “new traits,” of which some are often negative when the distance matrix is not Euclidian. To correct this, we used Cailliez's correction (Cailliez, 1983). Finally, we set each species as being weighted by their relative abundance.

The Rao index considers the sum of the pairwise distances between species weighted by their relative abundance (Botta‐Dukát, 2005). Although Mouchet et al. (2010) have indicated the index is actually a mix of functional richness and divergence, it can be converted into a pure metric of functional divergence by means of an adjustment with null models (Mason, De Bello, Mouillot, Pavoine, & Dray, 2013). Both the Rao and the Simpson index were computed using the function “RaoRel” and the Jost correction (i.e., Rao_corr_ = 1/(1 − index)) implemented in the R package cati (Jost, 2007; Taudiere, Violle, & Munoz, 2015). This Jost‐corrected index was used to derive equivalent communities, in which the total diversity would be equal to species richness if all species had the same abundance (for taxonomic). The same approach was applied to the Rao index, which allowed us to compare both taxonomic and functional facets with the same framework (see De Belo et al., 2010 for a detailed explanation of this procedure). Finally, we do not assign any weight to the traits. Thus, all traits have the same importance for functional indices.

### 2.4 Whole community and group‐specific patterns

Although our main aim was to test whether overall functional diversity in cacao agroforestry (regardless of species richness and/or composition) is lower than that observed in nearby mature forests, we also calculated all functional indices and the Simpson index for specific ecological groups. We suspected that some functional groups may suffer greater losses of functional diversity in cacao agroforestry, such as frugivores and understory insectivores (Faria et al., 2006). Thus, we repeated our analysis for four subsets of species: (a) forest specialists, (b) habitat generalists, and birds that contribute to (c) seed dispersal, or (d) invertebrate removal (mainly insectivores). We considered species with high and low forest dependency as being specialists and generalists, respectively, according to the classification of Birdlife International (2015). For the last two groups, we considered all the species that contains (a) fruits and/or seeds or (b) invertebrates in their diet, respectively. With regards to the inclusion of the term “granivores” in seed dispersers, Hulme (2002) lists several reasons for considering the term “frugivore–granivore” as being more appropriate with regards to the process of seed dispersal: (a) frugivorous vertebrates also consume seeds and (b) seed predators act in other facets of the seed dispersal cycle, such as interactions between plants ant their seed dispersers. Furthermore, our assignment of species as granivores was based on information in the literature on stomach contents, and thus, the presence of seeds may have been a consequence of fruit consumption.

### 2.5 Standardized effect size and null models

We used standardized effect size (SES) to correct any bias associated with the species richness for each functional diversity metric (Mason et al., 2013). We calculated the SES for the three functional indices (i.e., sesFD, sesFEve, and sesRao) as:

$$\text{SES}=\frac{\left(\text{observed index}-\text{mean expected values}\right)}{\text{standard deviation from the expected values}}$$

We generated 999 expected values for each index using the null model procedure of Swenson, Anglada‐Cordero, and Barone (2011). Treating each landscape as the regional pool of species we randomly reshuffled the row names (species names) on the trait matrix. Thus, the patterns of trait covariance, overall phenotypes, species occupancy rates, community species richness level, distributions of abundance, and species beta diversity were maintained.

In addition, calculating SES allowed us to draw inferences regarding the main assembly processes. If the SES > 0 (i.e., observed values of the indices are higher than expected by chance), this suggests that the community has less ecologically similar species. In the opposite situation (i.e., SES < 0), the community is structured by more functionally similar species. Coexistence of functionally similar species (positive SES) can indicate biotic pressures, such as competition, on community assembly (Mouchet et al., 2010; Mouillot et al., 2013). In contrast, negative SES can indicate that only species with traits determined by the environment can coexist at the regional scale, that is, niche filtering (Mouchet et al., 2010). To test whether these processes differ from the expected by chance (i.e., equal or close to zero), we performed one‐sample *t* tests (*μ* = 0).

### 2.6 Data analysis

We compared the mean values of richness, taxonomic Rao (Simpson), and functional SES‐indices between the forest fragments and cacao agroforestry in both landscapes using permutation *t* tests (100,000 iterations; perm package; Fay, 2010). Therefore, we tested whether mean differences between habitats differ from what would be expected by chance. To reduce the chance of false‐positive discoveries due to a large number of comparisons with the same data in each landscape (i.e., five comparisons per three indices in each landscape: 15 pairwise comparisons), we considered that solely *p*‐values <0.01 are “significant” and conclusive. We also explored the possibility that our results may be driven by spatial autocorrelation, given that some sites are spatially close to one another (Faria et al., 2007). According to the Moran's I index (ape package; Dutheil & Paradis, 2014), there was little evidence of spatial autocorrelation for functional and taxonomic metrics, with the exception of the Simpson index of insectivore communities in Una (*p* = 0.005; Supporting Information Appendix S2). Finally, we tested the independence between taxonomic and functional metrics using the Spearman correlation with assess whether the functional indices capture an effect different than what would be expected by taxonomic metrics. All analyses were run in R 3.1.3 (R Core Team, 2015).

## 3 RESULTS

### 3.1 Functional diversity measures

**Figure 3:**
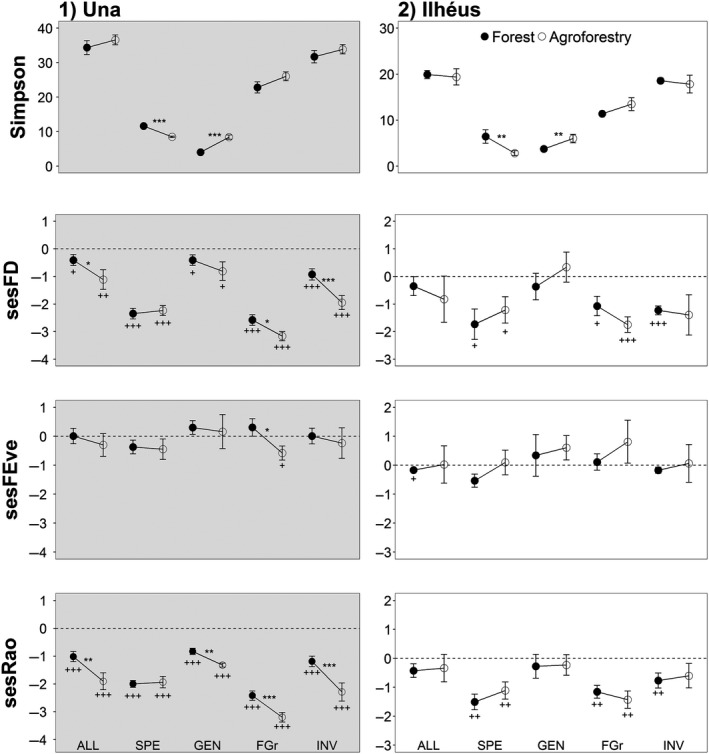
Simpson index and standardized effect size (SES, observed index – mean null models/*SD* null models) of FD, FEve and Rao functional metrics in the landscapes of Una (in gray) and Ilhéus, southern Bahia, Brazil. Values that are lower than expected by chance (i.e., <0, given that the *p*‐value <0.01) indicate a process of functional clustering, and the opposite situation indicates functional overdispersion. ALL: all bird species of the communities; FGr: species that contribute to seed dispersal (frugivores/granivores); GEN: habitat generalists; INV: species that contribute to invertebrate removal; SPE: forest specialist. The significance level of each pairwise comparison between habitats (*) and of the assembly processes (“+” below the bars) are indicated by the number of symbols, for example, *p* < 0.10 (*), *p* < 0.05 (**), and *p* < 0.01 (***)

We did not observe differences between bird communities from mature forests and cacao agroforestry for the entire community in both landscapes (Figure 3). Therefore, functional diversity differences of bird communities between both habitats did not differ from what would be expected by chance. Forest specialist and generalist birds showed the same results. However, we observed lower functional diversity in bird communities that contribute to seed dispersal (frugivores/granivores) and invertebrate removal (insectivores) in agroforestry of Una (the forested landscape, Figure 3). These decreases differed from what would be expected by chance (Figure 3). Thus, frugivores/granivores in agroforestry suffered declines in functional divergence (sesRao), while insectivores showed decreases in functional richness (sesFD) and divergence (Figure 3). We did not observe declines of functional evenness (sesFEve) for the entire community and for the other ecological groups in both landscapes. Furthermore, the Ilhéus landscape did not show evidence of functional differences between the habitats (Figure 3).

On average, functional divergence of frugivores/granivores and insectivores in agroforestry of Una was 1.32 and 1.92 times lower than in forests, respectively (Figure 3). Furthermore, forest insectivores in Una showed 2.10 times more functional richness than agroforestry ones (mean SES difference = 1.021; *p* < 0.001). These responses of functional diversity in agroforestry communities differed from those of taxonomic diversity. Despite the observed loss of functional diversity of seed dispersers and insectivores in Una agroforestry, bird communities in the agrosystem showed species richness greater than nearby forests, while the Simpson index did not show a difference between habitats (Table 1, Figure 3)

### 3.2 Correlation between functional and taxonomic indices

Functional metrics in both landscapes were not correlated with taxonomic ones (i.e., species richness and Simpson index). There was only one exception, the sesRao index that was correlated with the richness and the Simpson index of the forest frugivore/granivore in Una (*r* = −0.812, *p* < 0.01; see Supporting Information Appendix S3 for all correlation tests). Thus, the response of functional and taxonomic diversity (especially the species richness) is decoupled. This is evidenced by the Rao index, while the functional Rao tends to show higher mean values of diversity for the forests, its taxonomic version (Simpson) tends to show either an opposite pattern or no differentiation between habitats.

### 3.3 Standardized effect size and communities assembly

We observed functional clustering (SES < 0) in most communities of Una (Figure 3). This structuring process affected the functional divergence of the entire community and all other ecological groups in both habitats. In addition, forest specialists, frugivores/granivores and insectivores showed the same pattern for the functional richness. We did not observe any evidence of functional overdispersion, as well as no deterministic structuring pattern for functional evenness. Therefore, communities not structured by functional clustering show random assembly, whereby the observed functional structure does not differ from what would be expected by chance. With regards to the types of habitat, bird communities in the agroforestry of Una appeared to be more affected by functional clustering, because the mean SES values were generally further from zero (Figure 3).

In Ilhéus, we observed only functional clustering for frugivores/granivores in agroforestry (mean = −1.747 ± 0.561) and insectivores in forests sites (mean = −1.229 ± 0.317; Figure 3). This pattern affected only the functional richness component. For all other comparisons, communities showed random assembly.

## 4 DISCUSSION

Cacao agroforestry systems in southern Bahia appear to play a relevant role in the maintenance of functional diversity at the landscape scale, given that the overall bird communities, forest specialists, and habitat generalists were as diverse as nearby forests for at least two functional components (functional richness and evenness). Given that these findings were observed in small plantations (<100 ha) of a forested landscape, the conservation value of cacao systems to harbor functional diversity relies on the maintenance of a high level of forest cover in the landscape. Initial studies in southern Bahia have also emphasized similar conclusions for birds and bats species richness (Faria et al., 2006, 2007). In this context, our data support the idea that smallholder cacao production in forest‐dominated landscapes can actually reduce biodiversity loss (Clough et al., 2011), by conserving species‐rich communities and functional diversity. Of course, these systems also rely on a level of reduced management, such as a moderate cover of shading trees (Clough et al., 2011). However, we also observed the declines of functional diversity for species that contribute to two important ecosystem functions, seed dispersa and invertebrate removal. Therefore, even small cacao agroforestry does not substitute a mature forest.

Impacts of cacao agroforestry on bird functional diversity in forested landscapes seem to be specific, affecting particular sets of species instead of the entire community. We identified two ecological bird groups that appear to be more sensitive to agroforestry changes: seed dispersers and insectivores. However, taxonomic metrics did not show the same response for these groups, although there is evidence of decreases in species richness of understory insectivores and frugivores in cacao plantations (Faria et al., 2006; Van Bael et al., 2007). Thus, the maintenance of species richness of particular ecological groups and functional diversity components is decoupled. For instance, agroforestry that showed species‐rich communities of frugivores/granivores and insectivores compared with nearby forests also showed a lower functional richness and divergence. A likely explanation for these responses of bird diversity is related to a gradual loss of forest specialists followed by an increase in habitat generalists due to agricultural intensification before our data collection. In the 1980s, more than 400,000 ha of southern Bahia were converted into cacao plantations (Alger & Caldas, 1994), which created more fragmented and degraded landscapes. Given this, more sensitive species may have been lost, but the remaining ones persisted in the forests of Una, where there is less agriculture. This increase of generalist species (e.g., nonforest birds) in cacao agroforestry may have increased community richness while the decline of specialists may have led to declines in functional diversity for particular ecological groups instead of the entire bird community. Therefore, assessments of functional diversity based only on the overall community patterns may not reveal impacts in particular ecosystem functions, such as seed dispersal.

Frugivores and insectivores account for more than 70% of bird species in tropical agroforests and more than 80% in forest habitats worldwide (Tscharntke et al., 2008). Likewise, these groups are also predominant in cacao systems, such as in southern Bahia. Thus, we recommend conservation actions focused on these ecological groups in order to preserve their ecological functions in agricultural systems. Furthermore, these actions also provide important ecosystem services for the plantations. For instance, insectivorous birds reduce the foliar damage in cacao trees by controlling the abundance of arthropods (Cassano, Silva, Mariano‐Neto, Schroth, & Faria, 2016), while the frugivorous ones act on the regeneration and the stability of plant communities (Whelan et al., 2008), being important for the maintenance of shading trees diversity. Thus, farm owners should be informed about the economic benefits to agriculture when there is higher forest cover in their plantation systems. In fact, intensive farm management and landscape deforestation can reduce bird diversity in cacao farms by reducing food availability, such as plants with bird‐dispersed seeds (Greenberg, Bichier, & Angón, 2000). This decrease in resources can favor some species while excluding other ones, which is consistent with our findings of niche filtering structure, which environmental constraints (e.g., food shortage and microhabitat losses) “filter” species with suitable traits.

Communities that are functionally more similar than expected by chance (i.e., functional clustering) are frequently interpreted as an effect of niche filtering or trait filtering (Mouchet et al., 2010; Mouillot et al., 2013), whereby species with particular sets of traits cannot persist in the environment (Mouchet et al., 2010). However, interpretations with regards to this pattern using a trait‐based approach should be cautious. Kraft et al. (2015) argued that evidence of environmental filtering (another synonym for niche filtering) sensu stricto requires testing whether species can tolerate local abiotic conditions (e.g., soil and climate) in the absence of any neighbors (e.g., competitors and predators). In fact, our trait‐based data are limited with respect to structural characteristics and local abiotic conditions of the habitats at the sampled sites. However, there are reasons to expect niche filtering for bird communities in cacao agroforestry. The conversion of native forests into cacao agroforestry changes the vertical structure due to the removal of the understory, herbaceous layer management, epiphytic plants, lianas, as well as by canopy thinning (Rice & Greenberg, 2000; Sambuichi, 2006). Thus, several microhabitats are lost or changed, which can reduce the availability of resources (e.g., fruits and seeds of understory plants). Also, agroforestry structure and farm management can potentially alter local conditions, such as solar radiation input and leaf litter abundance, which again affect the availability of food items (e.g., fruits and soil insects). Finally, the functional divergence index can be interpreted as a measure of niche differentiation (according to Mason et al., 2005). On average, this functional metric in communities of agroforestry was lower compared with forests, which may support our idea that there are losses of resources even in the small agrosystems of a forested landscape.

Bird communities in agricultural landscapes may also show functional structures assembled by neutral processes, that is, observed functional diversity does not differ from what would be expected by chance. Thus, the functional structure will be an outcome of the dispersal rates (i.e., the order of arrival) and ecological drifts among species (i.e., reproduction and death; Hubbell, 2006; Hidasi‐Neto et al., 2012). In other words, communities are assembled according to the neutral theory (Hubbell, 2006), which predicts that species and individuals in the community are functionally equivalent (redundant). In fact, this may explain the response of functional evenness (abundance‐based functional component), which showed neutral assembly for all tested groups. Therefore, trait combinations in species and individuals in each community do not differ from those observed in the regional pool. This also emphasizes a pattern of high functional redundancy in our landscapes. Likewise, high redundancy and functional neutral assembly were also observed in bird communities in the Amazon (Hidasi‐Neto et al., 2012). In summary, niche filtering and neutral processes may structure bird communities at the local scale in forested landscapes, such as Una. In contrast, our findings in the cacao‐dominated landscape, Ilhéus, pointed out neutral processes as the main process structuring communities.

The dominance of cacao systems in the landscape seems to cause a high functional redundancy between forests and cacao agroforestry. This is also consistent with one of the main assumptions of the neutral theory (Hubbell, 2006). If a community is assembled by highly redundant species, declines in richness have little influence on functional diversity (Flynn et al., 2009). Furthermore, specific trophic groups also may show different patterns of trait redundancy compared with the entire community. Again, seed dispersers and insectivores appear to be more sensitive to agricultural intensification, given that these birds in Ilhéus suffered effects of niche filtering, although solely for functional richness. However, in this landscape, most of the ecological groups did not show any determinant structuring patterns. This may strongly suggest that the conversion of the forests into agrosystems has made the communities more similar and redundant due to gradual nonrandom species loss. Given this, it is likely that past environmental changes triggered a gradual process of functional homogenization in the small forest fragments surrounded by agricultural habitats. The replacement of specialists by generalists can generate functional homogenization (Clavel, Julliard, & Devictor, 2011), which may occur more strongly in the small forest patches.

In summary, our study identified more strong impacts on bird functional diversity when cacao agroforestry dominates the landscape, but also we observed positive impacts in agroforestry of a forested landscape. As suggested for taxonomic diversity, forest cover is a crucial factor for maintaining the functional diversity of birds in cacao systems (Clough et al., 2011; Faria et al., 2007). Finally, our study stresses the need for conservation efforts of seed dispersers and insectivores in order to harbor their ecosystem functions. Our findings corroborated in part with our hypotheses, given that we expected declines of functional diversity in the agroforestry, especially for forest specialists. Thus, smallholder cacao production can actually contribute to the creation of biodiversity‐friendly landscapes (Clough et al., 2011), which potentially combine species conservation, maintenance of ecosystem functions (e.g., carbon storage and seed dispersal), and food security.

## 5 CONCLUSIONS

Cacao agroforestry plays an important role in conserving bird diversity (taxonomic and functional) in forested landscapes. However, even smallholder agrosystems can lead to a decline in the functional diversity of frugivores/granivores and insectivores, affecting two ecosystem functions: seed dispersal and invertebrate removal. Furthermore, these declines are decoupled from those observed with taxonomic metrics. On the other hand, if agroforestry dominates the landscape, forest remnants may suffer strong functional homogenization. The better scenario where cacao systems can actually harbor species‐rich communities and ecosystem functions relies on small plantations with reduced farm management in a forested landscape.

## CONFLICT OF INTEREST

None declared.

## AUTHORS' CONTRIBUTIONS

JR, CGM, and SC conceived the ideas and designed methodology; RRL provided the bird lists and landscape information; JR and CGM organized the trait matrix; JR analyzed the data; JR and SC led the writing of the manuscript. All authors contributed critically to the drafts and gave final approval for publication.

## Data Availability

Data sources used to measure functional indices are cited in the text and Support Information. The main database (Handbook of the Birds of the World) can also be entirely accessed for a fee at www.hbw.com/species.
