# Supplementary material for: The conservation value of cacao agroforestry for bird functional diversity in tropical agricultural landscapes
Source: Ecol Evol. 2019 Jun 25;9(14):7903–13. doi: 10.1002/ece3.5021 (PMC6662317; doi:10.1002/ece3.5021)
Supplement: Supplementary file 1 [file ECE3-9-7903-s001.docx]

# The conservation value of cacao agroforestry for bird functional diversity in tropical agricultural landscapes

Joedison dos S. Rocha, Rudi Ricardo Laps, Caio Graco Machado and Sofia Campiolo

**Appendix S1.** Additional references used for traits matrix compilation.

**Body mass**

Birdlife International (2015) Datazone. Avaliable at http://www.birdlife.org/datazone (accessed 30 July 2016).

Magalhães, V.S., Azevedo-Júnior, S.M. De, Lyra-neves, R.M. De, Telino-Júnior, W.R. & Souza, D.P. De. (2007) Biologia de aves capturadas em um fragmento de Mata Atlântica, Igarassu, Pernambuco, Brasil. *Revista Brasileira de Zoologia*, **24**, 950–964.

Reinert, B.L., Pinto, J.C., Bornschein, M.R., Pichorim, M. & Marini, M.A. (1996) Body masses and measurements of birds from southern Atlantic Forest, Brazil. *Revista Brasileira de Zoologia*, **13**, 815–820.

Sick, H. (1997) *Ornitologia Brasileira* (2nd ed). Rio de Janeiro: Nova Fronteira.

**Foraging strata/substrate**

Machado, A.B.M., Drummond, G.M. & Paglia, A.P. (eds). (2008) *Livro vermelho da fauna brasileira ameaçada de extinção* (N.o 2). Belo Horizonte e Brasília: MMA/Fundação Biodiversitas.

Perlo, B.V. (2009) *A field guide to the birds of Brazil*. New York: Oxford University Press.

Ridgely, R. & Tudor, G. (2009) *Field guide to the songbirds of South America: the passerines*. Austin, TX: The Academy of National Sciences of Philadelphia, World Land Trust, and University of Texas Press.

Sick, H. (1997) *Ornitologia Brasileira* (2nd ed). Rio de Janeiro: Nova Fronteira.

WWF-Brazil. (2010) *Guia de aves Mata Atlântica Paulista – Serra do Mar e Serra de Paranapiacaba*. São Paulo: WWF-Brazil/Fundação Florestal do Estado de São Paulo.

**Food types**

Aguiar, K.M.O. & Coltro Jr, L.A. (2008) Dietas de algumas espécies de aves das famílias Thamnophilidae, Grallariidae e Formicariidae do Amapá. *Revista Brasileira de Ornitologia*, **16**, 376–379.

Ballarini, Y., Frizzas, M.R. & Marini, M.Â. (2013) Stomach contents of Brazilian non-passerine birds. *Revista Brasileira de Ornitologia*, **21**, 235–242.

Barros, R.A.M., Costa, C.A. & Pascotto, M.C. (2014) Diet and feeding behavior of the White-naped Jay, *Cyanocorax cyanopogon* (Wied, 1821) (Aves, Passeriformes, Corvidae) in a disturbed environment in central Brazil. *Brazilian Journal of Biology*, **74**, 899–905.

Cabral, J.D.C., Granzinolli, M.A.M. & Motta-Junior, J.C. (2006) Dieta do quiriquiri, *Falco sparverius* (Aves: Falconiformes), na Estação Ecológica de Itirapina, SP. *Revista Brasileira de Ornitologia*, **14**, 393–399.

Cestari, C. & Pizo, M.A. (2013) Frugivory by the White-bearded Manakin (*Manacus manacus*, Pipridae ) in restinga forest, an ecosystem associated to the Atlantic forest. *Biota Neotropical*, **13**, 345–350.

De Vasconcelos, M.F., Lopes, L.E. & Hoffmann, D. (2007) Dieta e comportamento de forrageamento de *Oreophylax moreirae* (Aves: Furnariidae) na Serra do Caraça, Minas Gerais, Brasil. *Revista Brasileira de Ornitologia*, **15**, 439–442.

Delarmelina, A.F.P. & Alves, M.A.S. (2009) Utilização de recursos alimentares por *Philydor atricapillus* e *P. rufum* (Aves: Furnariidae) em uma área de Mata Atlântica da Ilha grande, RJ. *Revista Brasileira de Ornitologia*, **17**, 59–64.

Draghi, J. & Alves, V.S. (2013) Dieta do tiê-sangue (*Ramphocelus bresilius* ) em uma área de restinga urbana no Sudeste do Brasil Diet of the Brazilian Tanager ( *Ramphocelus bresilius* ) in an urban shrubby restinga in Southeast Brazil. *Boletim do Museu Paraense Emílio Goeldi*, **8**, 449–457.

Durães, R. & Marini, M.A. (2005) A quantitative assessment of bird diets in the Brazilian Atlantic Forest , with recommendations for future diet studies. *Ornitologia Neotropical*, **16**, 65–83.

Faria, G.M.M. De & Passamani, M. (2013) Dieta da Coruja-da-Igreja (*Tyto alba*, Scopoli, 1769) no Sul de Minas Gerais e sua relação com disponibilidade de presas. *Revista Brasileira de Zoociências*, **15**, 247–252.

Fernandes, F.R., Cruz, L.D. & Rodrigues, A.A.F. (2007) Diet of the Gray-Breasted Martin (Hirundinidae: *Progne chalybea*) in a wintering area in Maranhão, Brazil. *Revista Brasileira de Ornitologia*, **15**, 436–438.

Filho, E.P.M.C., Canuto, M. & Zorzin, G. (2006) Biologia reprodutiva e dieta do gavião preto (*Buteogallus u. urubitinga*: Accipitridae) no sudeste do Brasil. *Revista Brasileira de Ornitologia*, **14**, 445–448.

Gaiotti, M.G. & Pinho, J.B. (2013) Diet of the Fuscous Flycatcher *Cnemotriccus fuscatus* (Wied, 1831) – Aves, Tyrannidae - in three habitats of the northern Pantanal, Mato. *Brazilian Journal of Biology*, **73**, 841–845.

Galetti, M. (1993) Diet of the Scaly-Headed Parrot (*Pionus maximiliani*) in a semideciduous forest in southern Brazil. *Biotropica*, **25**, 419–425.

Galetti, M. (1997) Seasonal abundance and feeding ecology of parrots and parakeets in a lowland Atlantic forest of Brazil. *Ararajuba*, **5**, 115–126.

Galetti, M., Laps, R., Pizo, M.A., Biotropica, S., Issue, S., Brazilian, T., Forest, A. & Galetti, M. (2013) Frugivory by Toucans (Ramphastidae) at Two Altitudes in the Atlantic Forest of Brazil. *Biotropica*, **32**, 842–850.

Gomes, V.S. da M., Loiselle, B.A. & Alves, M.A.S. (2008) Birds foraging for fruits and insects in shrubby restinga vegetation, southeastern Brazil. *Biota Neotropica*, **8**, 21–31.

Hoffmann, D., Vasconcelos, M.F., Lopes, L.E. & Rodrigues, M. (2007) Comportamento de forrageamento e dieta de *Polystictus superciliaris* ( Aves , Tyrannidae ) no sudeste do Brasil. *Ilheringia Séries Zoológicas*, **97**, 296–300.

Kristosch, G.C. & Marcondes-Machado, L.O. (2001) Diet and feeding behavior of the Reddish-bellied Parakeet (*Pyrrhura frontalis*) in an araucaria. *Ornitologia Neotropical*, **12**, 215–223.

Lima, A.L. de C., Manhães, M.A. & Piratelli, A.J. (2011) Ecologia trófica de *Conopophaga lineata* (Conopophagidae) em uma área de mata secundária no sudeste do Brasil. *Revista Brasileira de Ornitologia*, **19**, 315–322.

Lima, A.L.D.C. & Manhães, M.A. (2009) Hábitos alimentares de *Basileuterus culicivorus* (Aves: Parulidae) em uma área de Mata Atlântica secundária, sudeste do Brasil. *Biota Neotropica*, **9**, 137–143.

Lima, C.A., Siqueira, P.R., Gonçalves, R.M.M., De Vasconcelos, M.F. & Leite, L.O. (2010) Dieta de aves da Mata Atlântica: uma abordagem baseada em conteúdos estomacais. *Ornitologia Neotropical*, **21**, 125–138.

Lopes, L.E. (2005) Dieta e comportamento de forrageamento de *Suiriri affinis* e *S. islerorum* (Aves, Tyrannidae) em um cerrado do Brasil central. *Iheringia. Série Zoologia*, **95**, 341–345.

Lopes, L.E., Fernandes, A.M. & Marini, M.Â. (2005) Diet of some Atlantic Forest birds. *Ararajuba*, **13**, 95–103.

Manhães, M.A. (2003) Dieta de traupíneos (Passeriformes, Emberizidae) no Parque Estadual do Ibitipoca, Minas Gerais, Brasil. *Sér. Zool. Porto Alegre*, **93**, 59–73.

Manhães, M.A. (2007) *Ecologia Trófica de Aves de Sub-Bosque Em Duas Áreas de Mata Atlântica Do Sudeste Brasileiro*. PhD thesis, Universidade Federal de São Carlos, São Carlos.

Manhaes, M.A., Dias, M.M. & Lima, A.L.C. (2015) Feeding resource partitioning between two understorey insectivorous birds in a fragment of Neotropical cloud forest. *Brazilian Journal of Biology*, **75**, 176–183.

Marini, M.A. (1992) Foraging behavior and diet of the Helmeted Manakin. *The Condor*, **94**, 151–158.

Mestre, L.A.M., Cohn-haft, M. & Dias, M.M. (2010) Diet and Prey Availability of Terrestrial Insectivorous Birds Prone to Extinction in Amazonian Forest Fragments. *Brazilian Archives of Biology and Technology*, **53**, 1371–1381.

Mikich, S.B. (2002) A dieta frugívora de *Penelope superciliaris* (Cracidae) em remanescentes de floresta estacional semidecidual no centro-oeste do Paraná , Brasil e sua relação com *Euterpe edulis* (Arecaceae). *Ararajuba*, **10**, 207–217.

Motta-Junior, J.C. (2002) Diet of breeding tropical Screech-owls (*Otus choliba*) in southeastern Brazil. *Journal of Raptor Research*, **36**, 332–334.

Paranhos, S.J., Araújo, C.B. de & Marcondes-Machado, L.O. (2007) Comportamento alimentar do Periquito-de-encontro-amarelo (*Brotogeris chiriri*) no interior do estado de São Paulo, Brasil. *Revista Brasileira de Ornitologia*, **15**, 95–101.

Parrini, R. & Pacheco, J.F. (2014) Contribuição ao conhecimento das dietas fitófagas dos dois sanhaçus (*Thraupis cyanoptera* e *Thraupis ornata*) endêmicos da Mata Atlântica. *Atualidades Ornitológicas*, **177**, 40–45.

Pinheiro, C.E.G., Bagno, M.A. & Brandão, R.A. (2003) Diet and foraging behavior of the rufous-tailed jacamar (*Galbula ruficauda* , Galbulidae) in central Brazil. *Ararajuba*, **11**, 241–243.

Piratelli, A. & Pereira, M.R. (2002) Dieta de aves na região leste de Mato Grosso do Sul, Brasil. *Ararajuba*, **10**, 131–139.

Pizo, M.A. (2007) The relative contribution of fruits and arthropods to the diet of three trogon species (Aves, Trogonidae) in the Brazilian Atlantic Forest. *Revista Brasileira de Zoologia*, **24**, 515–517.

Pizo, M.A., Silva, W.R., Galetti, M. & Laps, R. (2002) Frugivory in cotingas of the Atlantic Forest of southeast Brazil. *Ararajuba*, **10**, 177–185.

Ragusa-Netto, J. & Fecchio, A. (2006) Plant food resources and the diet of a parrot community in a gallery forest of the southern Pantanal (Brazil). *Brazilian Journal of Biology*, **66**, 1021–1032.

Ramos, C.C. de, Benedito, E. & Zawadzki, C.H. (2011) Dieta e conteúdo calórico de aves atropeladas na região central do estado do Paraná, Brasil. *Biotemas*, **24**, 153–170.

Ranvaud, R., Freitas, K.C. de, Bucher, E.H., Dias, H.S., Avanzo, V.C. & Alberts, C.C. (2001) Diet of Eared doves (*Zenaida auriculata* , Aves, Columbidae ) in a sugar-cane colony in south-eastern Brazil. *Brazilian Journal of Biology*, **61**, 651–660.

Repenning, M., Basso, H.C.D.P., Rossoni, J.R., Krügel, M.M. & Fontana, C.S. (2009) Análise comparativa da dieta de quatro espécies de cucos (Aves: Cuculidae), no sul do Brasil. *Zoologia*, **26**, 443–453.

Roda, S.A. (2006) Dieta de *Tyto alba* na Estação Ecológica do Tapacurá, Pernambuco, Brasil. *Revista Brasileira de Ornitologia*, **14**, 449–452.

Rubim, P. (2009) Sazonalidade e dieta frugívora do saí-andorinha *Tersina viridis* (Illiger, 1911) em reflorestamento da mata ciliar do Rio Mogi Guaçu, São Paulo, Brasil. *Biota Neotropica*, **9**, 111–115.

Salvador-Junior, L.F. (2010) Behaviour and diet of the Mantled Hawk *Leucopternis polionotus* (Accipitridae; Buteoninae) during deforestation of an Atlantic Rainforest landscape in Southeast Brazil. *Revista Brasileira de Ornitologia*, **18**, 68–71.

Silva, F.H.A. (2007) *Dieta do gavião-real* Harpia Harpyja (Aves: Accipitridae*)*. Master degree thesis, Universidade Federal do Amazonas e Instituto de Pesquisas da Amazônia, Manaus, Brasil.

Simão, I., Santos, F.A.M. dos & Pizo, M.A. (1997) Vertical stratification and diet of psittacids in a tropical lowland forest of Brazil. *Ararajuba*, **5**, 169–174.

Siqueira, P., Vasconcelos, M.F., Gonçalves, R.M.M. & Leite, L.O. (2015) Assessment of stomach contents of some amazonian birds. *Ornitologia Neotropical*, **26**, 79–88.

Souto, G.H.O. (2010) *Ecologia alimentar de aves insetívoras de um fragmento de mata decídua do extremo norte da Mata Atlântica.* Master degree thesis, Universidade Federal do Rio Grande do Norte, Natal, Brasil.

Uejima, A.M.K., Boesing, A.L. & Anjos, L. Dos. (2012) Breeding and foraging variation of the Plush-Crested Jay (*Cyanocorax chrysops*) in the Brazilian Atlantic Forest. *The Wilson Journal of Ornithology*, **124**, 87–95.

Wischhoff, U., Marques-Santos, F. & Rodrigues, M. (2014) Foraging behavior and diet of the vulnerable Cinereous Warbling-finch *Poospiza cinerea* (Aves, Emberizidae). *Brazilian Journal of Biology*, **74**, 821–827.

Zaca, W., Silva, W.R. & Pedroni, F. (2006) Diet of the Rusty-margined Guan (*Penelope superciliaris*) in an altitudinal forest fragment of southeastern Brazil. *Ornitologia Neotropical*, **17**, 373–382.

Zilio, F. (2015) Dieta de *Falco sparverius* (Aves : Falconidae) e *Athene cunicularia* (Aves : Strigidae) em uma região de dunas no sul do Brasil. *Revista Brasileira de Ornitologia*, **14**, 379–392.

**Foraging method**

Fitzpatrick, J.W. (1980) Foraging Behavior of Neotropical Tyrant Flycatchers. *Condor*, **82**, 43-57.

Gabriel, V. de A. & Pizo, M.A. (2005) Foraging behavior of tyrant flycatchers (Aves, Tyrannidae) in Brazil. *Revista Brasileira de Zoologia*, **22**, 1072-1077.

Loss, A.C.C. & Silva, A.G. (2005) Comportamento de forrageio de aves nectarívoras de Santa Teresa – ES. *Natureza on line*, **3**, 48–52.

Machado, A.B.M., Drummond, G.M., Paglia, A.P. (eds). (2008) *Livro vermelho da fauna brasileira ameaçada de extinção*. N.o 2. MMA/Fundação Biodiversitas, Belo Horizonte e Brasília, Brazil.

Martins-Oliveira, L., Leal-Marques, R., Nunes, C.H., Franchin, A.G. & Marçal-Júnior, O. (2012) Forrageamento de *Pitangus sulphuratus* e de *Tyrannus melancholicus* (Aves: Tyrannidae) em hábitats urbanos. *Bioscience Journal*, **28**, 1038-1050.

Sick, H. (1997) *Ornitologia Brasileira*. 2nd ed. Nova Fronteira, Rio de Janeiro, Brazil.

Wiki Aves. (2016) A enciclopédia das aves do Brasil. Http://www.wikiaves.com.br [accessed March 2016].

WWF-Brazil. (2010) *Guia de aves Mata Atlântica Paulista – Serra do Mar e Serra de Paranapiacaba*. 1st ed. WWF-Brazil/Fundação Florestal do Estado de São Paulo, São Paulo.

**Activity period**

Sick, H. (1997) *Ornitologia Brasileira*. 2nd ed. Nova Fronteira, Rio de Janeiro, Brazil.

Wiki Aves. (2016) A enciclopédia das aves do Brasil. Http://www.wikiaves.com.br [accessed March 2016].

WWF-Brazil. (2010) *Guia de aves Mata Atlântica Paulista – Serra do Mar e Serra de Paranapiacaba*. 1st ed. WWF-Brazil/Fundação Florestal do Estado de São Paulo, São Paulo.

**Mixed-flocks**

Flores, F.M. (2014) *Composição e dinâmica de bandos mistos de aves da Mata Atlântica do Baixo Sul da Bahia, Brasil*. Master degree thesis, Universidade Estadual de Feira de Santana, Feira de Santana.

Aleixo, A. (1997) Composition of mixed-species bird flock and abundance of flocking species in a semideciduous forest of southeastern Brazil. *Ararajuba*, **5**, 11–18.

Maldonado-Coelho, M. & Marini, M.Â. (2003) Composição de bandos mistos de aves em fragmentos de Mata Atlântica no sudeste do Brasil. *Papéis Avulsos de Zoologia (MZUSP)*, **43**, 31–54.

Davis, D.E. (2007) A Seasonal Analysis of Mixed Flocks of Birds in Brazil. *Ecology*, **27**, 168–181.

Batista, R.O., Machado, C.G. & Miguel, R. dos S. (2013) A composição de bandos mistos de aves em um fragmento de Mata Atlântica no litoral norte da Bahia. *Bioscience Journal*, **29**, 2001–2012.

Develey, P.F. & Peres, C.A. (2000) Resource seasonality and the structure of mixed species bird flocks in a coastal Atlantic forest of southeastern Brazil. *Journal of Tropical Ecology*, **16**, 33–53.

Silva, J.N. da. (2010) Composição de bandos mistos de aves da Mata Atlântica da região serrana do estado do Espírito Santo, Brasil. *Atualidades Ornitológicas*, **155**, 12–15.

Machado, C.G. (1999) A composição dos bandos mistos de aves na Mata Atlântica da Serra de Paranapiacaba, no sudeste brasileiro. *Revista Brasileira de Biologia*, **59**, 75–85.

Ghizoni-Jr, I.R. & Azevedo, M.A.G. (2006) Composição de bandos mistos de aves florestais de sub-bosque em áreas de encosta e planície da Floresta Atlântica de Santa Catarina, sul do Brasil. *Biotemas*, **19**, 47–53.

Ghizoni-jr, I.R. (2009) Composição de bandos mistos de aves no Parque Estadual das Araucárias, oeste de Santa Catarina, Brasil. *Biotemas*, **22**, 143–148.

Brandt, C.S., Hasenack, H., Laps, R.R. & Hartz, S.M. (2009) Composition of mixed-species bird flocks in forest fragments of southern Brazil. *Zoologia*, **26**, 488–498.

Machado, C.G. & Rodrigues, N.M.R. (2000) Alteração de altura de forrageamento de espécies de aves quando associadas a bandos mistos. *Ornitologia Brasileira: perspectivas, conservação e pesquisa* (eds M.A.S. Alves, J.M.. Silva & V.M. Sluys), pp. 231–239. Ed. UERJ, Rio de Janeiro.
