# Supplementary material for: The conservation value of cacao agroforestry for bird functional diversity in tropical agricultural landscapes
Source: Ecol Evol. 2019 Jun 25;9(14):7903–13. doi: 10.1002/ece3.5021 (PMC6662317; doi:10.1002/ece3.5021)
Supplement: Supplementary file 2 [file ECE3-9-7903-s002.docx]

# The conservation value of cacao agroforestry for bird functional diversity in tropical agricultural landscapes

Joedison dos S. Rocha, Rudi Ricardo Laps, Caio Graco Machado and Sofia Campiolo

**Appendix S2** - Spatial Autocorrelation tests (Moran's I) for all tested datasets, i.e. total bird community (“All”), only forest specialist or habitat generalist (“Spe” and “Gen”, respectively), and birds with contribution to seed dispersal (“FGr”) and invertebrates removal (“Inv”), and for the taxonomic (richness and Simpson) and functional measures (sesFD, sesFEve, and sesRao).

| Landscape | Dataset | Richness | Simpson | sesFD | sesFEve | sesRao |
| --- | --- | --- | --- | --- | --- | --- |
| Una | All | -0.090 | 0.209 | -0.093 | 0.020 | -0.081 |
|  | P | 0.774 | **0.012** | 0.758 | 0.478 | 0.835 |
|  |  |  |  |  |  |  |
|  | Spe | -0.023 | -0.184 | -0.078 | -0.090 | -0.025 |
|  | P | 0.744 | 0.255 | 0.860 | 0.778 | 0.755 |
|  |  |  |  |  |  |  |
|  | Gen | -0.183 | -0.010 | 0.057 | 0.050 | -0.135 |
|  | P | 0.259 | 0.660 | 0.285 | 0.318 | 0.488 |
|  |  |  |  |  |  |  |
|  | FGr | -0.016 | 0.210 | -0.072 | -0.110 | -0.110 |
|  | P | 0.693 | **0.010** | 0.903 | 0.635 | 0.635 |
|  |  |  |  |  |  |  |
|  | Inv | -0.121 | 0.238 | -0.138 | -0.041 | -0.084 |
|  | P | 0.562 | **0.005** | 0.472 | 0.874 | 0.815 |
|  |  |  |  |  |  |  |
| Ilhéus | All | -0.267 | -0.154 | -0.138 | -0.100 | -0.030 |
|  | P | 0.152 | 0.896 | 0.955 | 0.575 | 0.215 |
|  |  |  |  |  |  |  |
|  | Spe | -0.304 | -0.311 | -0.069 | -0.251 | -0.038 |
|  | P | 0.040 | **0.018** | 0.402 | 0.216 | 0.204 |
|  |  |  |  |  |  |  |
|  | Gen | -0.270 | -0.145 | -0.169 | -0.025 | -0.104 |
|  | P | 0.111 | 0.976 | 0.773 | 0.135 | 0.670 |
|  |  |  |  |  |  |  |
|  | FGr | -0.293 | -0.134 | -0.178 | -0.162 | -0.068 |
|  | P | 0.084 | 0.887 | 0.667 | 0.829 | 0.417 |
|  |  |  |  |  |  |  |
|  | Inv | -0.241 | -0.161 | -0.212 | -0.148 | -0.018 |
|  | P | 0.254 | 0.819 | 0.342 | 0.945 | 0.178 |
